# Neurofilament light chain on intensive care admission is an independent predictor of mortality in COVID-19: a prospective multicenter study

**DOI:** 10.1186/s40635-023-00547-x

**Published:** 2023-09-28

**Authors:** Theodor Sievert, Ingrid Didriksson, Martin Spångfors, Gisela Lilja, Kaj Blennow, Henrik Zetterberg, Attila Frigyesi, Hans Friberg

**Affiliations:** 1 Department of Clinical Medicine, Anaesthesiology and Intensive Care, Lund University, Lund, SE-22185 Sweden; 2 Department of Intensive and Perioperative Care, Skåne University Hospital, Lund, SE-22185 Sweden; 3 Department of Intensive and Perioperative Care, Skåne University Hospital, Malmö, SE-20502 Sweden; 4 Department of Anaesthesia and Intensive Care, Kristianstad Hospital, Kristianstad, SE-29133 Sweden; 5 Department of Neurology, Skåne University Hospital, Lund, SE-22185 Sweden; 6 Department of Psychiatry and Neurochemistry, Institute of Neuroscience and Physiology, The Sahlgrenska Academy, University of Gothenburg, Mölndal, SE-43180 Sweden; 7 Clinical Neurochemistry Laboratory, Sahlgrenska University Hospital, Mölndal, SE-43180 Sweden; 8 Department of Neurodegenerative Disease, University College London Institute of Neurology, London, United Kingdom; 9 United Kingdom Dementia Research Institute, University College London, London, United Kingdom; 10 Hong Kong Center for Neurodegenerative Diseases, Hong Kong, China; 11 Wisconsin Alzheimer’s Disease Research Center, University of Wisconsin School of Medicine and Public Health, University of Wisconsin-Madison, Madison, United States of America

**Keywords:** Intensive care, Critical care, COVID-19, Biomarkers, Neurological injury, Neurofilament light chain, Glial fibrillary acidic protein, Tau, Outcomes, Mortality

## Abstract

**Background:**

Neurofilament light chain (NfL), glial fibrillary acidic protein (GFAP), and total-tau protein (tau) are novel blood biomarkers of neurological injury, and may be used to predict outcomes in critical COVID-19.

**Methods:**

A prospective multicentre cohort study of 117 consecutive and critically ill COVID-19 patients in six intensive care units (ICUs) in southern Sweden between May and November 2020. Serial NfL, GFAP and tau were analysed in relation to mortality, the Glasgow Outcome Scale Extended (GOSE) and the physical (PCS) and mental (MCS) components of health-related quality of life at one year.

**Results:**

NfL, GFAP and tau on ICU admission predicted one-year mortality with an area under the curve (AUC) of 0.82 (95% confidence interval [CI] 0.74$-$0.90), 0.72 (95% CI 0.62$-$0.82) and 0.66 (95% CI 0.54$-$0.77). NfL on admission was an independent predictor of one-year mortality (*p *= 0.039). Low NfL and GFAP values were associated with good PCS ($\ge$45) at one year but not with good MCS ($\ge$45) or GOSE ($\ge$5).

**Conclusions:**

NfL on ICU admission was an independent predictor of mortality. High levels of NfL, GFAP and tau were associated with mortality but not with poor GOSE in survivors at one year. Low levels of NfL and GFAP were associated with improved physical health-related quality of life.

**Graphical Abstract:**

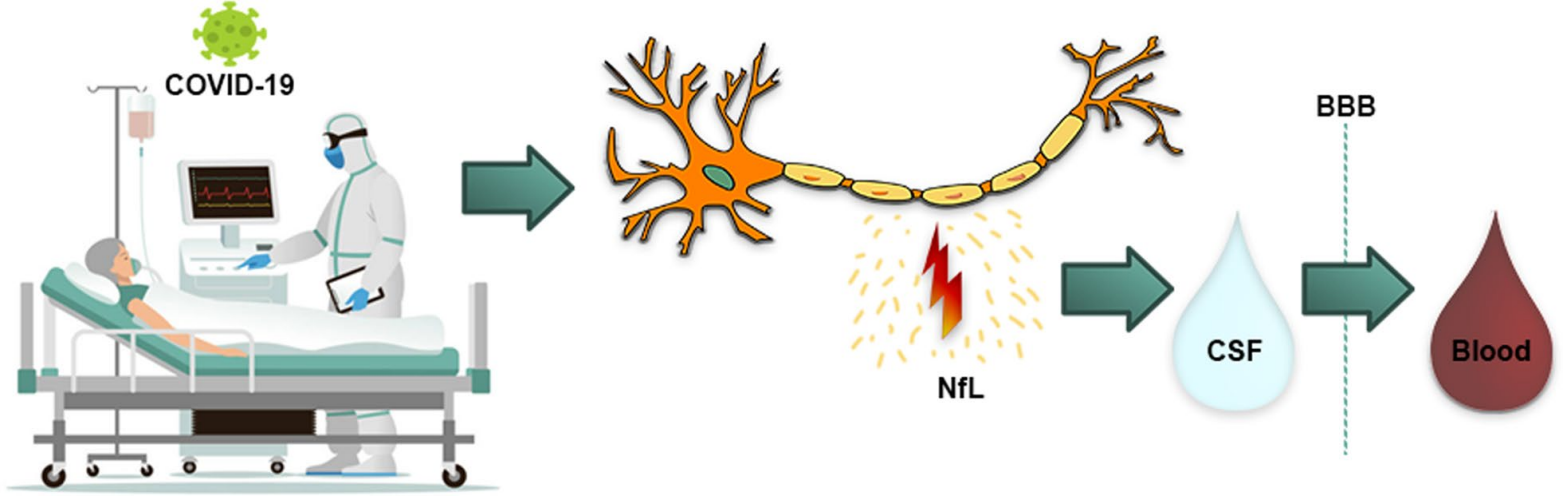

## Background

### Critical COVID-19

Critical COVID-19 requiring intensive care and ventilatory support is characterised by acute respiratory distress syndrome (ARDS) [1] and multiorgan dysfunction, posing a significant risk of severe morbidity and mortality [2, 3]. Identifying patients likely to develop critical COVID-19 can aid resource allocation and facilitate adequate therapeutic interventions. The search for predictive biomarkers and clinical features that can forecast the disease course may be particularly important in COVID-19 research.

**Fig. 1:**
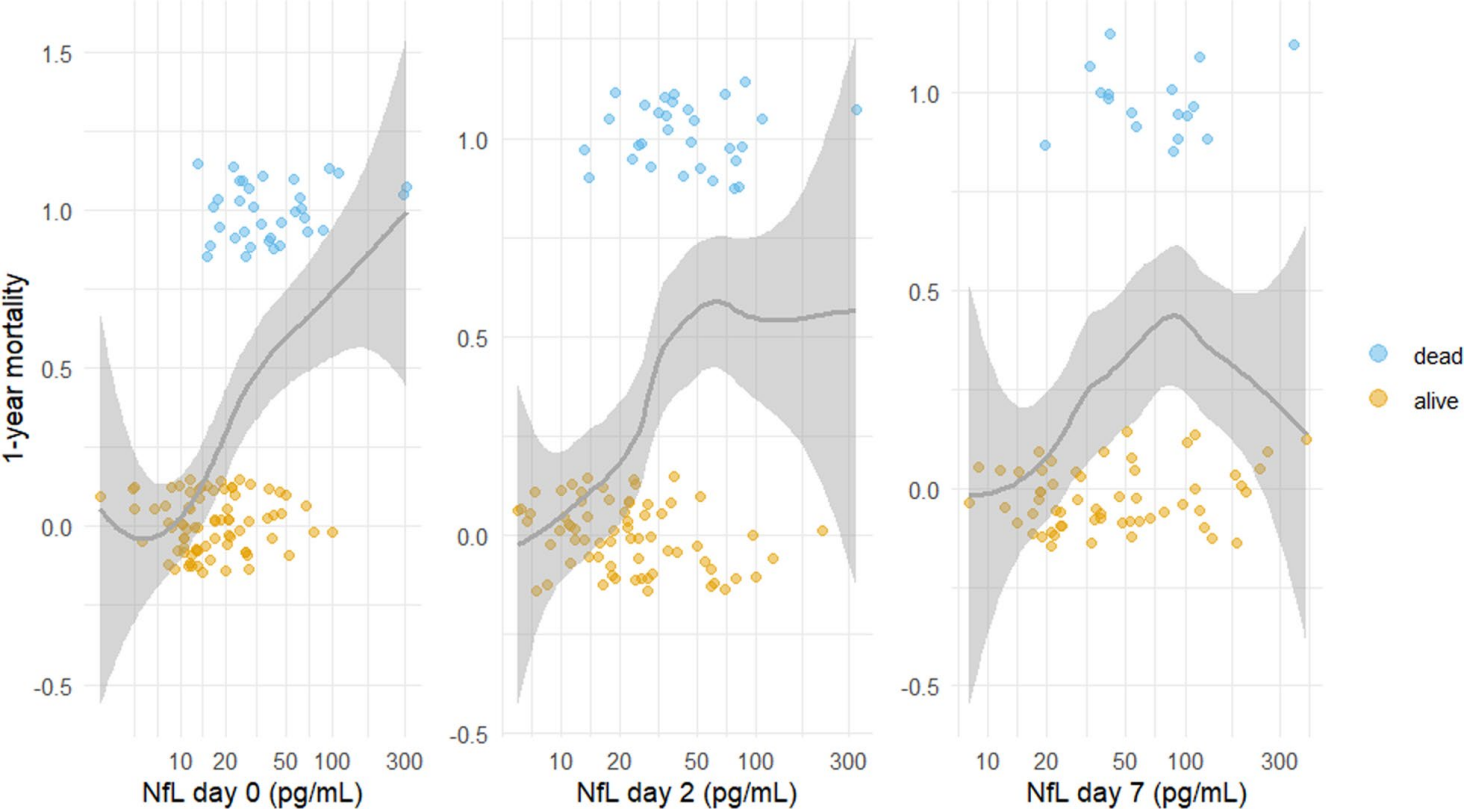
Mortality as a function of neurofilament light chain (NfL) on ICU admission in critical COVID-19. The grey line (with a grey 95% confidence band) is a local polynomial regression of mortality. For clearer visualisation, patients who survived beyond one year (mortality of 0) are indicated as having a mortality range of $-$0.15 to 0.15. In contrast, patients who did not survive one year (mortality of 1) are shown as having mortality in the range of 0.85 to 1.15

**Fig. 2:**
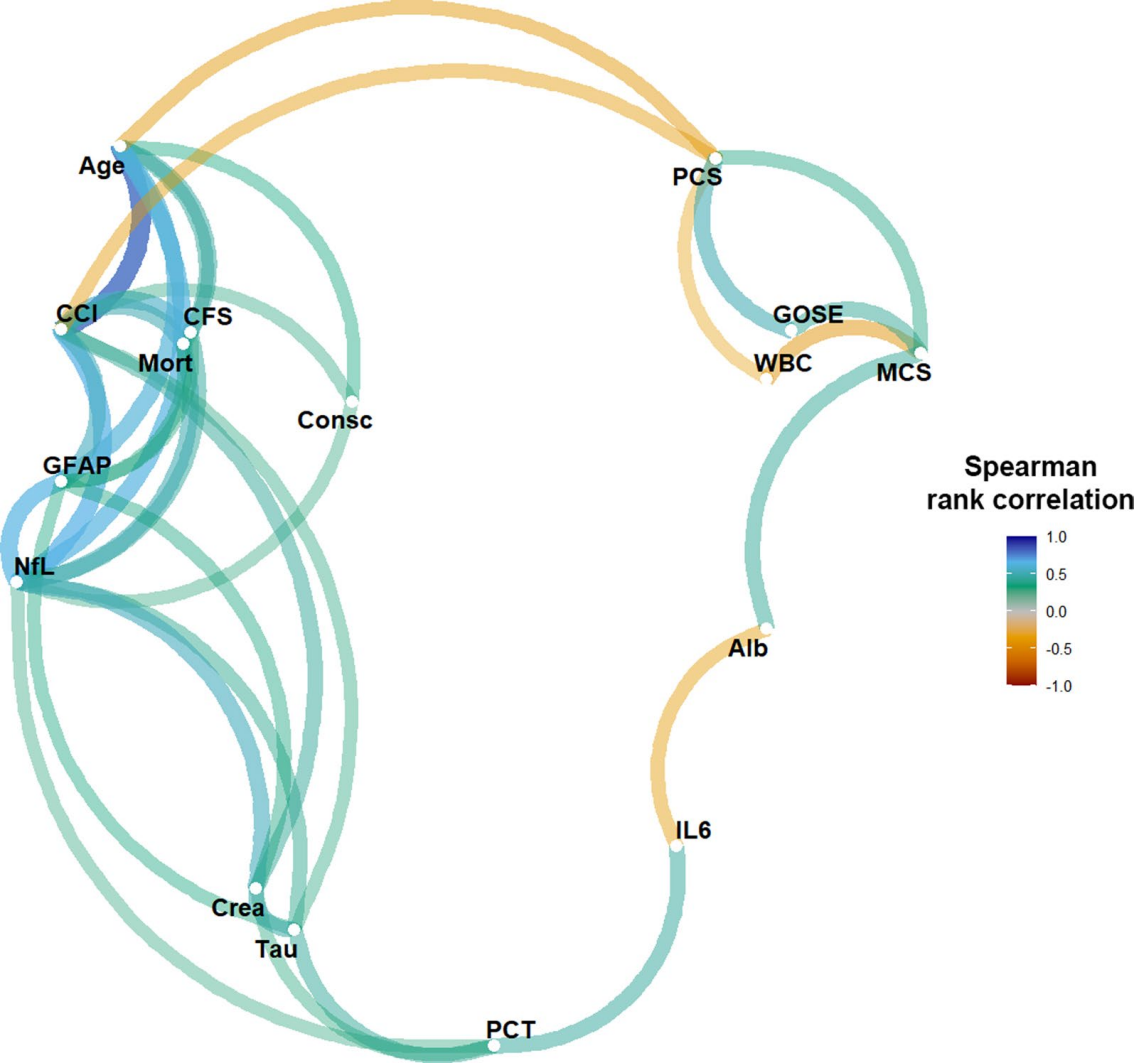
Correlation network for NfL, GFAP, and tau. The correlation network visualises pairwise correlations between the variables by colouring the edges with the Spearman rank correlation. The deeper the colour, the stronger the correlation. Only variables connected (directly or indirectly) with NfL, GFAP, or tau through absolute correlations > 0.3 are visualised. The spatial arrangement of the variables (nodes) groups them into strongly correlated clusters of variables. Biomarker blood samples were collected on admission to the intensive care unit (ICU). *Alb* serum albumin, *CCI* Charlson comorbidity index, *CFS* clinical frailty scale *Consc* altered consciousness pre-ICU, *Crea* serum creatinine, *GFAP* serum glial fibrillary acidic protein, *GOSE* Glasgow Outcome Scale Extended,* Mort* one-year mortality, *IL6* serum interleukin-6, *MCS* short Form-36 Item Questionnaire Health Survey Version 2 mental component summary,* NfL* serum neurofilament light chain, *PCT* serum procalcitonin, *PCS* short Form-36 Item Questionnaire Health Survey Version 2 physical component summary, *WBC* white blood cell count

### Neurological complications of COVID-19

Sepsis-associated encephalopathy (SAE) is a frequent early finding in sepsis, and long-term effects can be severe [4]. Sepsis may induce disruption of the blood–brain barrier (BBB) and may also cause cerebral hypoperfusion [5]. Similar mechanisms have been proposed in COVID-19 [6, 7]. SARS-CoV-2 infection has been associated with neurological complications [8], and the severity of COVID-19 seems to be an important determinant [9].

Post-mortem studies have found evidence of SARS-CoV-2 in the brain tissue of patients who eventually succumbed to COVID-19, suggesting a direct viral invasion targeting neurons via angiotensin-converting enzyme 2 receptors [10, 11]. Moreover, the extensive systemic inflammatory response in critical COVID-19, sometimes resulting in a “cytokine storm”, can also lead to neurological injury [12].

**Fig. 3:**
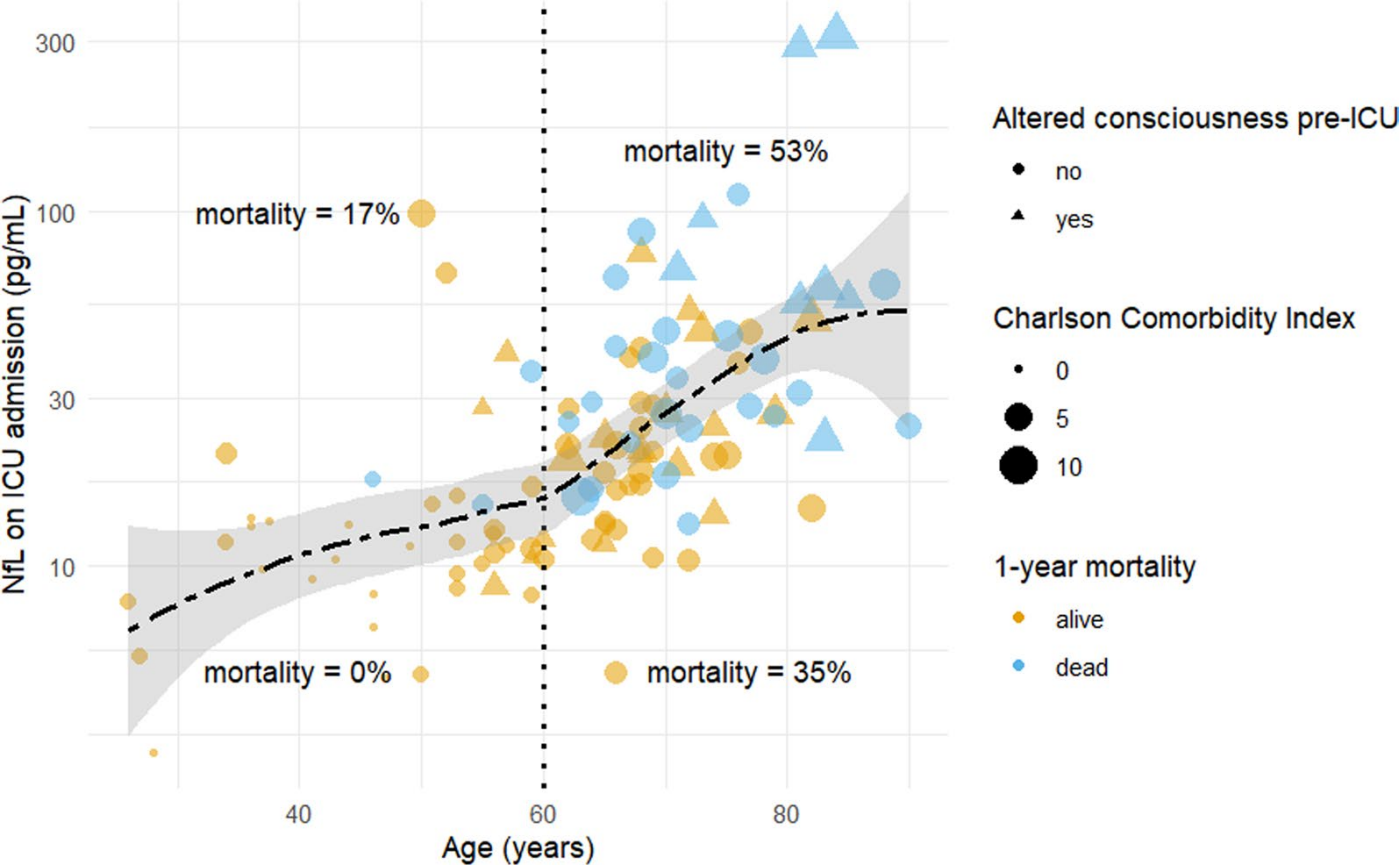
NfL as a function of age and mortality in critical COVID-19. The dash-dotted horizontal line is a local polynomial regression line. The dotted vertical line represents the age of 60. The local polynomial regression lines and vertical lines divide data into sectors with one-year mortality displayed for each sector. Altered consciousness before ICU admission was defined as GCS verbal response <4 after the onset of COVID-19 symptoms and before admission to the intensive care unit. *GCS* Glasgow Coma Scale, *ICU* intensive care unit, *NfL* serum neurofilament light chain

**Fig. 4:**
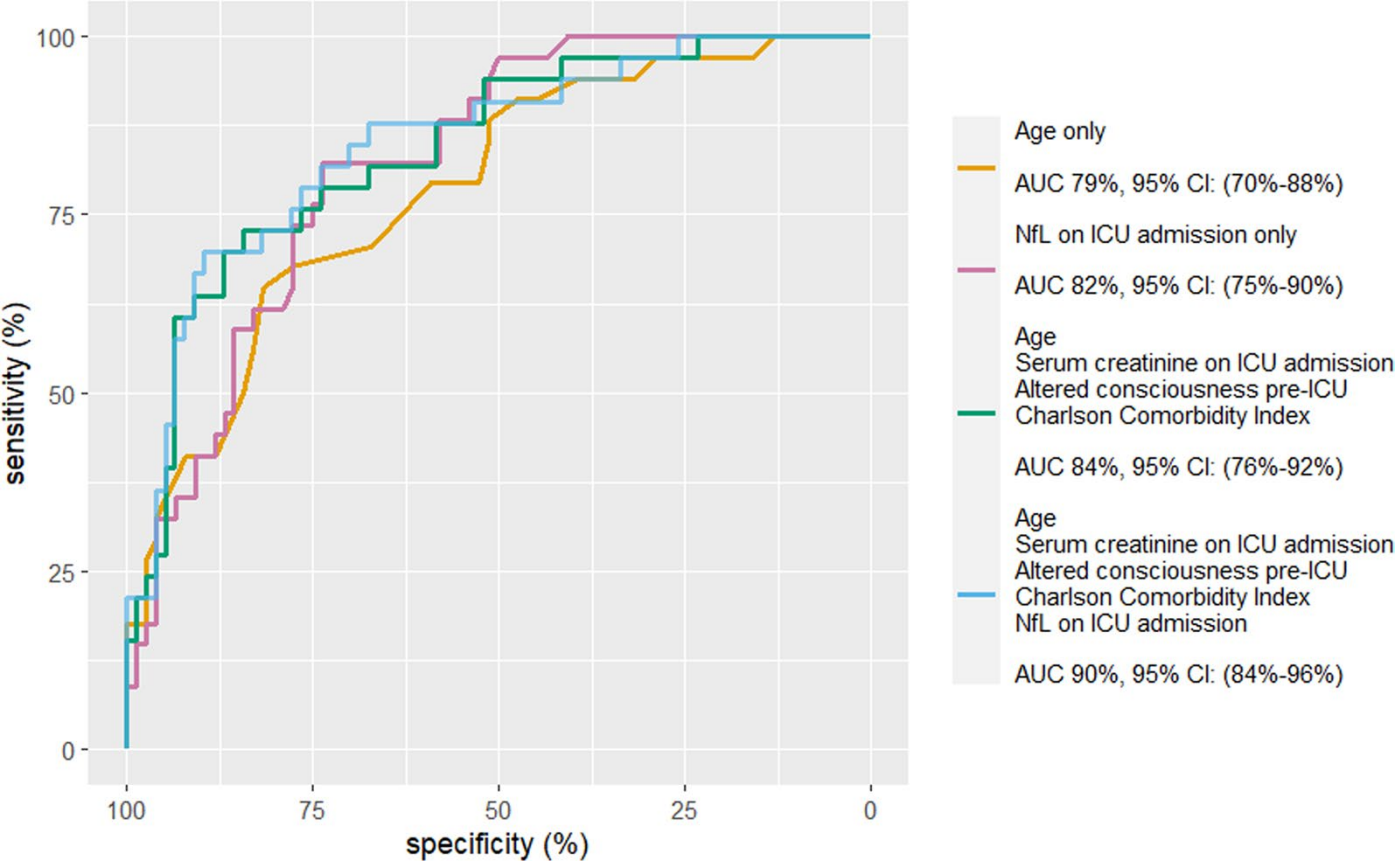
Receiver operator characteristic (ROC) curves and their corresponding areas under the curve (AUC) for one-year mortality prediction in critical COVID-19. All the models were based on logistic regression. The difference between the model using age, serum creatinine on ICU admission, altered consciousness before ICU admission and Charlson comorbidity index and the model using age, serum creatinine on ICU-admission, altered consciousness, Charlson comorbidity index and NfL on ICU-admission was significant (p = 0.029). *ICU* intensive care unit, *NfL* serum neurofilament light chain

### Biomarkers of CNS injury

Neurofilament light chain (NfL) is a 68-kDa structural protein found in neurons, and elevated levels in cerebrospinal fluid and blood have been associated with various neurological conditions [13, 14]. The normal levels of NfL in the blood increase with age and have been reported to vary from lows around 3 pg/ml in younger adults to highs exceeding 50 pg/ml for those above 65 years of age [15, 16].

Glial fibrillary acidic protein (GFAP) is a type III intermediate filament protein in astrocytes [17]. Increased levels of GFAP have been associated with reactive gliosis, a typical response to central nervous system (CNS) injury, and have been detected in various neurological diseases [18, 19]. Normal blood levels of GFAP are also age-dependent, with values well below 100 pg/ml in young adults, rising fourfold for >65 year-olds [20].

Total-tau protein, commonly referred to as tau, is another neuronal protein that has received considerable attention in the context of Alzheimer’s disease [21]. Further, elevated blood levels of tau have been associated with the appearance of encephalopathy in septic patients [22]. Normal tau levels in the blood are in the 1.8$-$2.7 pg/ml range in functionally intact older adults [23].

Elevated NfL and GFAP blood levels have been reported in COVID-19 [24], and higher levels are associated with severe disease. NfL is most extensively studied and high NFL levels are associated with worse clinical outcomes in critical COVID-19 [25]. Health-related quality of life (HrQoL) has been insufficiently investigated in critical COVID-19. The physical and mental component scores of SF-36v2®have shown long-lasting deterioration in hospitalised COVID-19, particularly in women and in those aged 41–60 years [25, 26].

This study aimed to describe associations between three biomarkers of CNS injury, NfL, GFAP, and tau, and one-year mortality as well as recovery at one year using the GOSE and the physical (PCS) and mental (MCS) components of SF-36v2®. We hypothesised that increased levels of all three biomarkers, particularly NfL, were associated with worse clinical outcomes in critical COVID-19.

## Methods

### Study design

This prospective multicentre cohort study is a part of the SweCrit COVID-19 study [2]. ClinicalTrials.gov identifier: NCT04974775. Surviving participants were invited to a follow-up one year after ICU admission, performed primarily face-to-face but could be replaced by a telephone interview. Certified interpreters were used when participants were deemed non-fluent in the Swedish language.

An age-matched control group was created by collecting blood samples and basic demographic data from healthy volunteers.

The manuscript was prepared per the Strengthening the Reporting of Observational Studies in Epidemiology (STROBE) guidelines [27].

### Participants

Critically ill patients, 18 years or older, with laboratory-confirmed SARS-CoV-2 infection were included at six intensive care units (ICU) in the Skåne Region, Sweden, between May 11, 2020, and November 30, 2020. The SweCrit COVID-19 study was predetermined to stop inclusion after one year (May 10 2021), and this cohort thus represents an initial pilot study. Patients were excluded if COVID-19 was not the primary cause of ICU admission.

### Variables

In survivors, functional outcome was assessed one year after admission using the clinician-reported Glasgow Outcome Scale Extended (GOSE), an ordinal scale ranging from 1 to 8, where 1 represents death and 8 a full recovery [28]. A GOSE $\ge 5$ was considered a good functional outcome.

To investigate HrQoL, the patient-reported SF-36v2® was used [29]. The SF-36v2® was calculated and presented as two summary scores of physical (Physical Component Summary, PCS) and mental (Mental Component Summary, MCS) HrQoL. Both PCS and MCS are presented as *T* scores with a range of 0–100, with lower scores indicating a worse HrQoL and 50 representing the mean value of a US normative population. PCS and MCS scores $\ge 45$ are considered normal or good HrQoL.

Altered consciousness before ICU admission was defined as no verbal response, incomprehensible sounds, or inappropriate words (Glasgow Coma Scale [GCS] verbal response <4) at any point after the onset of COVID-19 symptoms and prior to admission to the intensive care unit.

### Data sources

Background and survival data were extracted from the patient administrative system for intensive care units (PASIVA) and the regional quality register COVID-IR. PASIVA is synchronised with the Swedish population register, containing survival data. Data regarding altered consciousness before ICU admission were gathered retrospectively through electronic medical records.

### Measurement of NfL, GFAP, and tau

Serial blood samples used to analyse NfL, GFAP, and tau were collected on ICU admission, day 2, and day 7 after ICU admission. Samples were centrifuged, aliquoted, frozen, and stored in the SWECRIT biobank at Region Skåne (BD-47, SC-1922). Samples collected later than 6 h after ICU admission were excluded. If the sampling time was missing, samples were included in the analysis if the freezing time was within 6 h. The frozen plasma samples were transported to the Clinical Neurochemistry Laboratory in Mölndal. Batch analyses of NfL, GFAP, and tau were performed on thawed samples using a commercially available single-molecule array (Simoa) method on an HD-X analyser according to the instructions from the manufacturer (Quanterix, Billerica, United States of America). Frozen plasma samples from the control group were analysed simultaneously. All analyses were performed by board-certified laboratory technicians at the Clinical Neurochemistry Laboratory, University of Gothenburg, Sweden, blinded to clinical data. Intra-assay coefficients of variation were around 5%.

### Statistics

#### Generals

For all hypotheses tests, we considered *p*-values <0.05 significant. To assess a difference in the location of two independent variables, we used the Wilcoxon rank-sum test (Mann–Whitney *U* test). Differences in proportions were assessed using Pearson’s $\chi ^2$ test.

#### Correlation network analysis

The pairwise Spearman rank correlation was calculated and visualised for absolute correlations >0.3 using the network_plot function of the corrr package in R [30, 31].

#### Local polynomial regression

Local polynomial regression [32] was used to visualise mortality as a function of the biomarkers using the default settings (loess) of geom_smooth of the ggplot2 package in R [30].

#### Regression models

Multivariable binary logistic regression was used to analyse one-year mortality, GOSE, and SF-36v2®. Areas under the curve (AUC) were derived from the receiver operating characteristic (ROC) curves [33]. Differences in AUCs were tested with the method of DeLong et al. [34]. The biomarkers were analysed using the base-10 logarithm.

## Results

### Participants

A total of 117 COVID-19 patients requiring intensive care were included. Healthy controls (*n *= 119) were used for comparison. Age was similarly distributed with a median age (25th and 75th percentiles) of 66 (56–72) years for the COVID-19 group and 65 (61–71) years for the healthy and age-matched controls.

### Biomarker levels and outcomes

The median levels (25th and 75th percentiles) of NfL, GFAP and tau on ICU admission were 20.4 (11.9$-$34.3) pg/mL, 176.0 (102.5$-$243.3) pg/mL and 7.64 (3.87$-$14.23) pg/mL, and were significantly higher in one-year non-survivors compared to survivors for all three biomarkers; see Table 1.

**Table 1:** Demographics and outcomes for the study population subdivided into one-year survivors and non-survivors

	**COVID-19**	**Non-survivors**	**Survivors**	*p*-value
*Number (% of population)*	117 (100)	35 (30)	82 (70)	
*Demography*				
Age (years)	66 (56–72)	71 (67–80)	62 (52–68)	<0.001
Female sex, *n* (%)	27 (23)	7 (20)	20 (24)	0.78
Body mass index	29.7 (26.2–34.8)	28.2 (24.6–31.6)	31.2(27.0–35.8)	0.031
Smoker ever, *n* (%)	54 (46)	18 (51)	36 (44)	0.73
*Comorbidities*				
Immunosuppressive therapy, *n* (%)	9 (8)	5 (14)	4 (5)	0.12
Systemic steroids (before COVID-19), *n* (%)	12 (10)	7 (20)	5 (6)	0.039
Charlson comorbidity index	3 (1–4)	4 (3–6)	2 (1–3)	<0.001
Clinical frailty scale	3 (2–4)	3 (2–5)	3 (2–3)	0.0064
Hypertension, *n* (%)	64 (55)	23 (66)	41 (50)	0.1
Complicated diabetes, *n* (%)	19 (16)	6 (17)	13 (16)	1
COPD and severe asthma, *n* (%)	25 (21)	12 (34)	13 (16)	0.047
Chronic kidney disease, *n* (%)	3 (3)	2 (6)	1 (1)	0.21
*Organ dysfunction and illness severity*				
SAPS 3 score	52 (46–63)	62 (53–70)	49 (43–57)	<0.001
Altered consciousness pre-ICU, *n* (%)	27 (23)	8 (23)	19 (23)	1
Systolic blood pressure (mmHg)	123 (110–136)	120 (100–134)	125 (111–137)	0.16
Temperature (mmHg)	37.6 (37–38.3)	37.3 (36.7–38.0)	37.8 (37.2–38.6)	0.011
P/F ratio Day 1 (min) (kPa) (mmHg)	12 (10–15)	13 (10–15)	12 (9–15)	0.51
PaCO_2_ Day 1 (max) (kPa) (mmHg)	5.3 (4.8–6.3)	5.3 (4.6–6.3)	5.2 (4.8–6.3)	0.93
pH	7.45 (7.40–7.49)	7.45 (7.37–7.49)	7.46 (7.41–7.49)	0.70
*Biochemistry*				
Creatinine (μmol/L)	79 (68–100)	95 (73–146)	75 (65–96)	0.002
Leukocytes (x10^9^/L)	8.9 (6.6–11.8)	9.3 (8.1–14.6)	8.4 (6.5–10.6)	0.12
Platelets (x10^9^/L)	214 (165–312)	185 (132–222)	230 (180–322)	0.0042
CRP (mg/L)	145 (89–186)	155 (99–183)	135 (89–190)	0.68
PCT (μg/L)	0.46 (0.2–0.95)	0.6 (0.37–0.89)	0.39 (0.15–0.98)	0.046
Lactate (mmol/L)	0.5 (0.2–0.9)	2.1 (1.6–3.2)	2.0 (1.5–2.5)	0.42
LD (μkat/L)	8.8 (6.4–11.0)	9.7 (7.9–12.8)	7.9 (6.3–10.8)	0.14
D-Dimer (mg/L)	1.45 (0.77–3.08)	1.45 (0.76–3.45)	1.5 (0.83–3.02)	0.97
Ferritin (μg/L))	1256 (611–2222)	1335 (979–2470)	1204 (555–2173)	0.24
IL-6 (ng/L)	86 (43–179)	93 (70–227)	81 (36–162)	0.20
Bilirubin (μmol/L)	8 (6–13)	11 (7–15)	8 (6–12)	0.12
NfL (pg/mL)	20.4 (11.9–34.3)	34.7 (24.7–60.0)	14.4 (10.7–22.8)	<0.001
GFAP (pg/mL)	176.0 (102.5–243.3)	215.0 (170.0–342–8)	135.5 (92.0–216.8)	<0.001
Tau (pg/mL)	7.64 (3.87–14.23)	11.95 (5.84–18.5)	5.61 (3.20–10.6)	0.0064

	**COVID-19**	**Non-survivors**	**Survivors**	*p*-value
*Clinical interventions and complications*				
Invasive mechanical ventilation, *n* (%)	61 (52)	22 (63)	39 (48)	0.10
Invasive mechanical ventilation^*^ (hours)	269 (144–508)	326 (188–548)	247 (131–494)	0.31
Prone position, *n* (%)	82 (70)	21 (60)	61 (74)	0.19
Tracheotomy, *n* (%)	23 (20)	6 (17)	17 (21)	0.80
CRRT, *n* (%)	16 (14)	4 (11)	12 (15)	0.78
ECMO, *n* (%)	3 (3)	2 (6)	1 (1)	0.21
Pulmonary embolism, *n* (%)	13 (11)	3 (9)	10 (12)	0.75
Cardiac arrest, *n* (%)	3 (3)	3 (9)	0 (0)	0.04
*Outcomes*				
ICU length of stay (days)	11 (5–24)	10 (5–21)	12 (5–27)	0.75
Mortality ICU, *n* (%)	22 (19)	22 (63)	NA	NA
Mortality hospital, *n* (%)	31 (26)	31 (89)	NA	NA
One-year mortality, *n* (%)	35 (30)	35 (100)	NA	NA
One-year GOSE	7 (6–8)	NA	7 (6–8)	NA
One-year GOSE$\ge 5$^**^, *n* (%)	52 (88)	NA	52 (88)	NA
One-year PCS	43 (38–49)	NA	43 (38–49)	NA
One-year PCS$\ge 45$^**^, *n* (%)	20 (63)	NA	20 (63)	NA
One-year MCS	50 (39–56)	NA	50 (39–56)	NA
One-year MCS$\ge 45$^**^, *n* (%)	29 (44)	NA	29 (44)	NA

Results are expressed as numbers with percentages or medians (with 25th and 75th percentiles). Biomarker blood samples were collected upon the patient’s arrival at the intensive care unit. *COPD* chronic obstructive pulmonary disease, *CRP* serum C-reactive protein, *CRRT* continuous renal replacement therapy, *ECMO* extracorporeal membrane oxygenation, *GFAP* serum glial fibrillary acidic protein, *GOSE* Glasgow Coma Scale Extended, *ICU* intensive care unit, *IL-6* serum interleukin-6, *LD* lactate dehydrogenase, *MCS* defined as short form-36 item Questionnaire Health Survey version 2 mental component summary, *NfL* serum neurofilament light chain, *PaCO2* arterial partial pressure of carbon dioxide, *PCS* defined as short Form-36 item Questionnaire Health Survey version 2 physical component summary, *PCT* serum procalcitonin, *P/F* ratio defined as PaO2 (arterial partial pressure of oxygen)/FiO2 (fraction of inspired oxygen), *SAPS 3* Simplified Acute Physiology Score III. *Only for patients with invasive mechanical ventilation. **Only for patients completing this functional outcome

In healthy controls, the corresponding levels for NfL, GFAP and tau were 12.0 (9.0$-$15.9) pg/mL, 110.0 (79.8$-$154.5) pg/mL and 6.57 (5.27$-$7.80) pg/mL. Levels differed significantly between critical COVID-19 and healthy controls for NfL (*p* $\le 0.0001$) and GFAP (p $\le 0.0001$) but not for tau.

Hospital mortality was 26% and one-year mortality was 30%. Most included patients had severe ARDS with a median *P*/*F* ratio of 12 kPa on day 1. More than half, 52%, required invasive mechanical ventilation. Age, Charlson comorbidity index, clinical frailty scale, SAPS-3 score, creatinine and platelets on admission differed significantly between survivors and non-survivors. There was no significant difference in gender distribution between survivors and non-survivors, while body mass index was significantly lower in non-survivors (Table 1).

### Univariate predictions of mortality, GOSE, and SF-36v2®

**Fig. 5:**
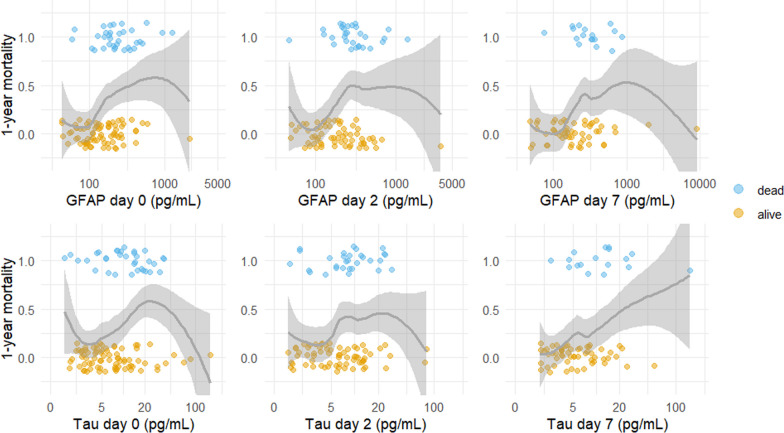
Mortality as a function of glial fibrillary acidic protein (GFAP) and tau on ICU admission in critical COVID-19. The grey line (with a grey 95% confidence band) is a local polynomial regression of mortality. For clearer visualisation, patients who survived beyond one year (mortality = 0) are indicated as having mortality in the range of $-$0.15 to 0.15. In contrast, patients who did not survive one year (mortality = 1) are shown as having mortality in the range of 0.85 to 1.15. *GFAP* serum glial fibrillary acidic protein

One-year mortality versus NfL on day 0 (admission), day 2 and day 7 is presented in Fig. 1. Higher NfL levels, particularly on day 0, were associated with increased one-year mortality. Higher GFAP and tau levels were also associated with increased one-year mortality; see Appendix, Fig. 5.

**Fig. 6:**
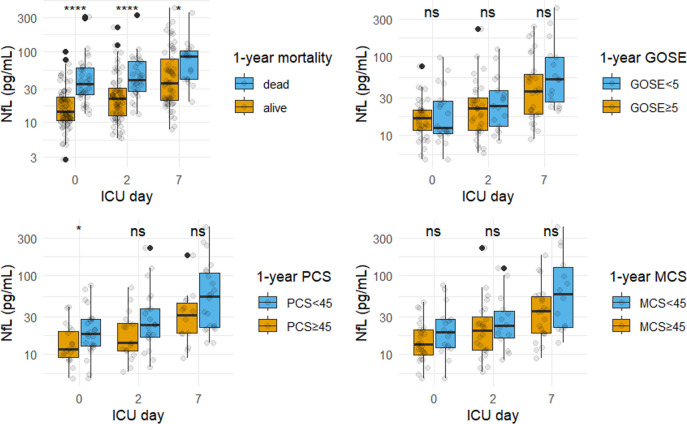
NfL levels for one-year outcomes mortality, GOSE, PCS and MCS in critical COVID-19. The Wilcoxon rank-sum test was used to assess the difference in NfL levels between dead and alive. *GOSE* Glasgow Outcome Scale Extended, *ICU* intensive care unit, *NfL* serum neurofilament light chain, *MCS* defined as short Form-36 item Questionnaire Health Survey version 2 mental component summary, *PCS* defined as short Form-36 item Questionnaire Health Survey version 2 physical component summary, *ns: p* $> 0.05$ * p $\le 0.05$ p $\le 0.01$ *** p $\le 0.001$ **** p $\le 0.0001$

**Fig. 7:**
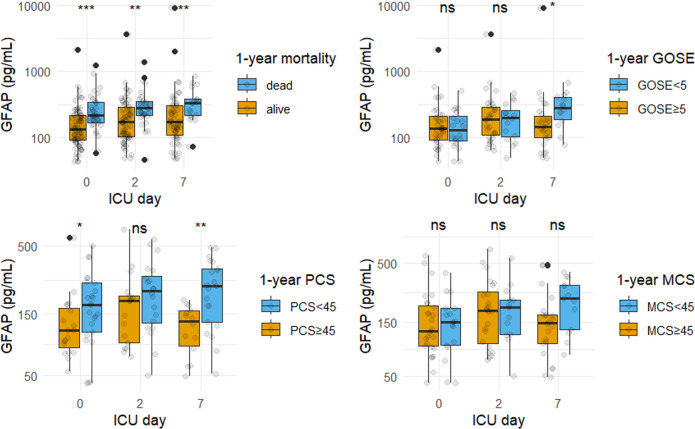
GFAP levels for one-year outcomes mortality, GOSE, PCS and MCS in critical COVID-19. The Wilcoxon rank-sum test was used to assess the difference in GFAP levels between dead and alive. *GFAP* serum glial fibrillary acidic protein, *GOSE* Glasgow Outcome Scale Extended, *ICU* intensive care unit, *MCS* defined as short Form-36 item Questionnaire Health Survey version 2 mental component summary, *PCS* defined as short Form-36 item Questionnaire Health Survey version 2 physical component summary, *ns: p* $> 0.05$ *p $\le 0.05$ *p* $\le 0.01$ ***p $\le 0.001$ ****p $\le 0.0001$

**Fig. 8:**
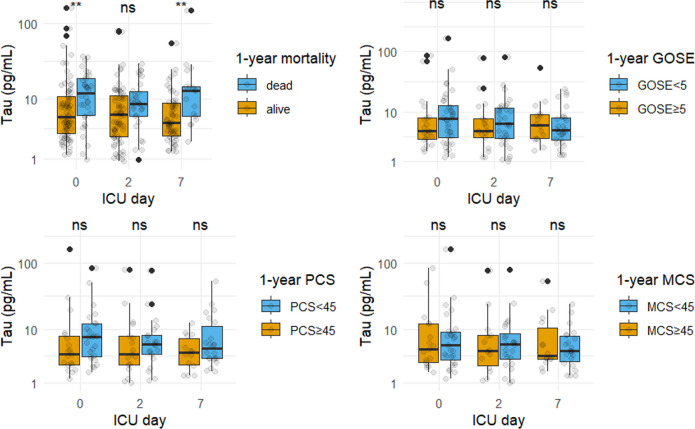
Tau levels for one-year outcomes mortality, GOSE, PCS and MCS in critical COVID-19. The Wilcoxon rank-sum test was used to assess the difference in tau levels between dead and alive. *GOSE* Glasgow Outcome Scale Extended, *ICU* intensive care unit, *MCS* defined as short Form-36 item Questionnaire Health Survey version 2 mental component summary, *PCS* defined as short Form-36 item Questionnaire Health Survey version 2 physical component summary, *ns: p* $> 0.05$ *p $\le 0.05$ *p* $\le 0.01$ ***p $\le 0.001$ ****p $\le 0.0001$

NfL levels on days 0, 2, and 7 for one-year survivors versus non-survivors, good or poor GOSE, PCS, and MCS, are presented in the Appendix, Fig. 6. NfL levels for days 0 (*p* $\le 0.0001$), 2 (*p* $\le 0.0001$), and 7 (*p* $\le 0.05$) were all significantly higher in non-survivors. NfL levels for day 0 (*p* $\le 0.05$) were significantly higher in patients with poor PCS. Corresponding analyses for GFAP and tau are presented in the Appendix, Figs. 7 and 8. GFAP levels for days 0 (*p* $\le 0.001$), 2 (*p* $\le 0.01$), and 7 (*p* $\le 0.01$) were all significantly higher in non-survivors. GFAP levels for days 0 (*p* $\le 0.05$) and 7 (*p* $\le 0.01$) were significantly higher in patients with poor PCS. Tau levels for days 0 (*p* $\le 0.01$) and 7 (*p* $\le 0.01$) were significantly higher in non-survivors. No significant differences in tau levels were observed in patients with poor PCS. Except for day 7 GOSE GFAP levels (*p* $\le 0.05$), no significant differences in biomarkers were observed for good versus poor outcomes in GOSE or MCS.

NfL on admission predicted one-year mortality with an AUC of 0.82 (95% confidence interval [CI] 0.74$-$0.90); see Fig. 4. GFAP and tau on admission predicted one-year mortality with an area under the curve (AUC) of 0.72 (95% CI 0.62$-$0.82) and 0.66 (95% CI 0.54$-$0.77), respectively.

### The correlations of NfL, GFAP and tau

**Fig. 9:**
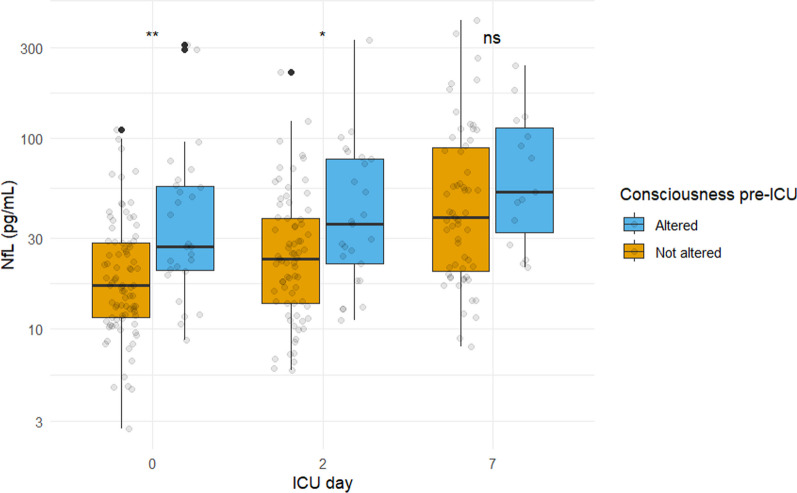
NfL levels on ICU-admission for altered consciousness before ICU-admission. Altered consciousness before ICU admission was defined as a GCS verbal response <4 after the onset of COVID-19 symptoms and before admission to the intensive care unit. The Wilcoxon rank-sum test was used to assess the difference in NfL levels between dead and alive. *GCS* Glasgow Coma Scale, *ICU* intensive care unit, *NfL* serum neurofilament light chain, *ns: p* $> 0.05$ *p $\le 0.05$ *p* $\le 0.01$ ***p $\le 0.001$ ****p $\le 0.0001$

To find significant clinical correlations, the correlations (Spearman rank) of NfL, GFAP and tau and the variables presented in Table 1 are visualised in Fig. 2 for absolute correlations > 0.3. Variables with no correlation to NfL, GFAP and tau were removed. NfL correlated with altered consciousness admission before ICU admission, as presented in Fig. 9.

### Multivariate predictions of mortality

Figure 3 shows age-adjusted levels for critical COVID-19. For patients younger than 60 years of age and NfL less than the age-adjusted levels, mortality was 0%, whereas, for NfL above the age-adjusted levels, mortality was 17%. Similarly, for patients older than 60 years and NfL lower than for the age-adjusted levels, the mortality was 35%, whereas, for NfL above the age-adjusted levels, the mortality was 53%.

Age, creatinine, CCI, and altered mental status before ICU admission generated an AUC for one-year mortality of 0.84 (95%CI 76–92%). Adding NfL on ICU admission to the model increased the AUC to 0.90 (95%CI 84–96%) (*p* = 0.028), showing that NfL on admission was an independent predictor of one-year mortality; see Fig. 4. GFAP and tau on admission were not predictors of one-year mortality (*p* = 0.11 and *p* = 0.17). No biomarkers were predictors of the functional outcomes or HrQoL (GOSE, MCS or PCS).

## Discussion

The main finding of this study is that NfL on ICU admission is a strong independent predictor of mortality in critical COVID-19.

For a better understanding of the role of NfL, GFAP and tau on ICU admission in critical COVID-19, a correlation network was created, revealing that NfL correlates primarily with age, renal function, CCI, CFS, altered consciousness before ICU admission, serum procalcitonin (PCT), and indirectly with interleukin-6 (IL-6) and albumin, apart from being correlated with GFAP and tau. GFAP and tau, consequently, had similar correlation patterns.

It is well-established that NfL increases with age and that age is a strong predictor of intensive care outcomes in COVID-19—in fact, better than the ICU gold standard Simplified Acute Physiology Score 3 (SAPS-3) [2]. Due to the correlation analyses and previously described associations of NfL, the multivariable logistic regression analyses of mortality included age, creatinine, CCI, and altered consciousness before ICU admission. GFAP and tau were also predictors of one-year mortality in critical COVID-19, albeit not independent predictors.

In univariate analyses, HrQoL in survivors showed that levels of NfL and GFAP on ICU admission differed for good versus poor PCS at one year, while no differences were seen for good versus poor MCS. Levels of tau did not differ for PCS or MCS at one year. There is no clear explanation for these differences, but our results are in line with those of Needham et al. [25].

The findings that NfL, GFAP and tau on ICU admission, i.e. before lengthy intensive care, showed predictive capabilities of one-year mortality in critical COVID-19 may suggest (1) a direct effect of SARS-CoV-2 on the CNS in critical COVID-19, (2) that they measure (possibly sub-clinical) pre-existing degenerative CNS disease, or (3) that they reflect a CNS effect of the secondary organ failure (e.g. hypoxia or hypoperfusion). These three possible explanations may also be viewed in light of a proposed increased BBB permeability in critical COVID-19 [6, 7].

Since levels of NfL in the cerebrospinal fluid (CSF) are much higher than in blood, initial studies primarily focused on analysing the CSF in neurological disease. The degree to which the BBB and blood–CSF barrier permeability influence the blood NfL levels is unclear. Explorative work used the CSF and serum albumin ratio to estimate BBB permeability. However, this ratio is rather a marker of the blood–CSF barrier [35, 36]. Patients with the highest CSF-to-serum albumin ratio also had the highest CSF and blood NfL levels, suggesting that an altered permeability of the blood–CSF barrier contributes to serum NfL [37, 38]. This relationship, however, was not observed in all studies [37–40]. Of note, there is an increased disruption of the BBB with ageing which may contribute to the increased levels of blood NfL in age-related diseases [41]. The increase in blood NfL levels with age may be driven by an increasing burden of comorbidities rather than the ageing process, such as the disruption of the BBB. Blood NfL levels have also been reported to depend on renal function and sex [42, 43].

The NfL and GFAP levels in critical COVID-19 in this study are within previously suggested reference intervals in healthy individuals [15, 16, 20]. Compared to previous COVID-19 studies, however, biomarker levels in the present study, especially NfL, are similar [24, 25]. NfL and GFAP levels in our age-matched controls were also significantly lower than in critical COVID-19. The previously reported slow release of NfL compared to GFAP is clearly shown in Figs. 6 and 7 [24], which also impacts absolute levels. Blood samples were collected within a relatively short time frame in the ICU and NfL levels in critical COVID-19 have been reported to normalise over time, up to 6 months later [24]. Our samples were collected at the defined start of ICU care, corresponding to an average 11 days after the onset of symptoms [2]. The differing levels of NfL and GFAP thus seem to adequately reflect health versus critical disease.

The precise cause of elevated NfL values in non-survivors cannot be determined in the present study. We have, however, corrected for comorbidities using the Charlson comorbidity index, making a pre-existing degenerative disease a less likely cause.

We speculate that the most likely cause for NfL elevation on admission in critical COVID-19, particularly in non-survivors, reflects a secondary CNS effect due to organ failure (hypoxia or hypoperfusion and consequent CNS injury) and recommend further studies to investigate this hypothesis in critical disease. If our hypothesis is correct, NfL could be used to evaluate intensive care in general, e.g. different sedation or drug strategies, blood pressure or ventilatory targets, using a biomarker reflecting perhaps the most important endpoint of intensive care—preserving cognitive abilities and emotional well-being.

### Limitations

The most important limitation of our study is the small sample size, as it limits our search for a more precise cause for the higher biomarker levels of CNS injury in non-survivors.

### Strengths

This study presents a prospectively and carefully studied population of critically ill COVID-19 patients. Serial biomarkers of CNS injury were collected over time and batch analysed together with healthy and age-matched controls, allowing for correct comparisons. We also allowed for corrections for renal function, comorbidities, and altered consciousness prior to admission. The detailed and mainly face-to face follow-up, including GOSE and HrQoL as measured by SF-36v2®one year after critical disease, is also a strength.

## Conclusion

NfL, GFAP, and tau on ICU admission for critical COVID-19 are predictors of one-year mortality. NfL is an independent (age, creatinine, comorbidities, altered consciousness before ICU admission) predictor of one-year mortality and may be a potential outcome predictor in critical disease in general. In addition, NfL and GFAP are associated with good physical HrQoL at 1 year.

## Data Availability

The datasets generated and analysed during the current study are not publicly available due to limitations in the ethical approval of the study and data management policies of Region Skåne. However, they are available from the corresponding author upon reasonable request.

## Appendix

See Figs. 5, 6, 7, 8, and 9.

## Abbreviations

ARDS: Acute respiratory distress syndrome
AUC: Area under the curve
BBB: Blood–brain barrier
CNS: Central nervous system
CI: Confidence interval
CSF: Cerebrospinal fluid
GCS: Glasgow Coma Scale
GFAP: Glial fibrillary acidic protein
GOSE: Glasgow Outcome Scale Extended
HrQoL: Health-related quality of life
ICU: Intensive care unit
IL-6: Interleukin-6
NfL: Neurofilament light chain
MCS: SF-36v2® mental component summary
PASIVA: Patient administrative system for intensive care units
PCT: Procalcitonin
PCS: SF-36v2® physical component summary
ROC: Receiver operating characteristic
SAPS 3: Simplified Acute Physiology Score III
SAE: Sepsis-associated encephalopathy
STROBE: Strengthening the Reporting of Observational Studies in Epidemiology
